# Farmers’ Perception of Fall Armyworm (*Spodoptera frugiperda*) as an Invasive Pest and Its Management

**DOI:** 10.3390/insects16040427

**Published:** 2025-04-18

**Authors:** Waseem Akbar, Sumaira Yousaf, Muhammad Farhan Saeed, Wafa A. H. Alkherb, Asim Abbasi, Nazih Y. Rebouh, Nazia Suleman

**Affiliations:** 1 Plant Protection Division, Nuclear Institute for Agriculture and Biology, Jhang Road, P.O. Box 128, Faisalabad 38000, Pakistan; 2 Nuclear Institute for Agriculture and Biology, College (NIAB-C), Pakistan Institute of Engineering and Applied Sciences (PIEAS), Islamabad 45650, Pakistan; 3 Plant Protection Division, Nuclear Institute of Agriculture, Tando Jam 70050, Pakistan; 4 Department of Environmental Sciences, COMSATS University Islamabad, Vehari Campus, Vehari 61100, Pakistan; 5 Department of Biology, College of Science, Qassim University, P.O. Box 6666, Buraidah 51452, Saudi Arabia; 6 Department of Entomology, University of Agriculture, Faisalabad 38040, Pakistan; asimuaf95@gmail.com; 7 Department of Environmental Management, Institute of Environmental Engineering, RUDN University, 6 Miklukho-Maklaya St., Moscow 117198, Russia

**Keywords:** maize, farming, larva, pest management, questionnaire, socio-economic, season, Pakistan

## Abstract

This study was carried out with the aim of understanding how farmers perceive and deal with fall armyworm (FAW) infestations in maize crops. Data based on a questionnaire were collected across nine districts of the Punjab province in Pakistan. Findings revealed that about 38% of farmers had medium-sized landholdings and had been cultivating maize crop for the last 11–20 years. Hybrid maize seed was used by 55% of the farmers, and 60% of the maize grown was used as fodder, as well as a cash crop. Surprisingly, only 39% of farmers were able to correctly identify FAW; however, the majority (72%) recognized the larvae as the most damaging stage. Although most of the farmers (71%) grew maize crops during both seasons, only 34% of the farmers recognized autumn as the peak infestation period of FAW. However, despite limited awareness, a high percentage (86%) of farmers managed FAW effectively using various chemical treatments. This study also highlighted the influence of some factors on farmers’ perception of FAW such as: age, farming experience, and maize cultivation practices. Overall, the findings emphasize the need for increased awareness of the basic biology and targeted management strategies for FAW to safeguard maize crops in the region.

## Simple Summary

This study investigated farmers’ perceptions and management of fall armyworm (FAW) pest in maize crop across nine districts of Punjab, Pakistan. The results showed that farmers generally had medium-sized farms, with experience of growing maize over ten years. The majority of the farmers grow maize as fodder, as well as a cash crop, availing both spring and autumn seasons. A few famers have better ability to identify FAW and consider autumn the peak infestation period of FAW. Despite limited awareness, most of the farmers recognized larvae as the most damaging stage and managed FAW using chemical treatments. The current study highlighted the need for better awareness of farmers regarding biology of FAW and also the understanding regarding its effective management strategies.

## 1. Introduction

*Spodoptera frugiperda* (J.E. Smith) fall armyworm (FAW) has been documented as a notorious pest of agricultural crops since 1797 [1]. The larval instars of FAW feed and damage a wide range of monocot and dicot plant families encompassing more than three hundred and fifty plant species. Its larvae mostly damage the cultivated grasses, with maize as their preferred host plant [2]. Historically, this pest is native to the American regions [3] and has been considered a major pest in the Western Hemisphere [4], causing significant damage to crops. However, a sudden outbreak in the African continent since early 2016 and further rapid spread to other countries [5] made FAW an alarming multi-continent menace for maize [6]. Consequently, it poses a serious threat to the global food security and livelihood of many farmers [7]. Reasons for heavy crop losses by FAW include its gregarious feeding, polyphagous nature, strong flying ability (Noctuidae family) and invasive nature [8].

The larvae can cause direct damage to maize plants by feeding on leaves, ears and cobs [9]. However, indirect damage to crops is mostly associated with photosynthetic disruption by defoliation [10]. Moreover, the FAW infested grains support fungal growth, which further deteriorates grain quality [11], causing pre-harvest losses. Recently, estimated yield losses in various crops by FAW larvae had been documented, amounting to 13 billion US dollars annually in African countries [12,13], whereas maize losses had been reported to reach 300–500 million US dollars or more during occasional peak outbreaks of FAW across different regions. These losses include direct or indirect maize yield reduction, poor quality grain production, heavy tolls for pest management and high costs associated with international trade due to legal bindings, such as phytosanitary protocols [14].

Maize is recognized as queen of cereal crops primarily due to its low cost of production and high productivity [15]. The higher yield potential of maize warrants enhanced income for farmers. Moreover, promising weather of Pakistan and two cropping seasons made maize the most preferred crop among the farming community. Maize has been valued as it provides food to man, feed to animal and raw material to industry [16]. In Pakistan, maize is placed among top three major cereal crops of the country, after wheat and rice. During the period 2018–2023, maize crop area was increased by almost 25% [17]. The preference of hybrid maize and expansion in its cultivated area are mainly due to its higher output return compared to other crops [18]. Early reports of some researchers confirmed the presence of FAW in Pakistan during 2019 [19,20]. Since then, farmers have been facing many socio-economic and agricultural problems in tackling this invasive pest, threatening the national economy and farmers’ income [13].

Usually, farmers rely on their indigenous information and knowledge to manage pests like FAW [13]. Such an approach, based on traditional knowledge, along with no or poor awareness and education of farmers, limited resources and marginalized returns seems to be the main hurdle in adoption of latest pest management strategies [21]. Furthermore, at the policy level, data regarding farmers’ perception, knowledge, their needs, and the suitability of pest management tactics are either unavailable or ignored by experts while devising pest management protocols [22]. This further exacerbates the overall situation relating to pest management.

Knowledge means understanding of farmers about FAW identification, life cycle and favorable ecological conditions, whereas information about damage symptoms of FAW and its possible management options are considered as perception [23]. Different countries have different ecological conditions, farming community approaches [24], regional cropping patterns and agronomic practices [7]. These diversified characteristics demand area-specific surveys to understand the problem of invasive FAW and its severity. In Pakistan, data regarding FAW management protocols among farmers is scarce or unavailable. Therefore, the basic data about FAW’s awareness and perception of the farming community in the country is much needed to devise a practical and sustainable management policy.

Keeping in view the above facts, this study was designed to gather information regarding farmers’ perception and knowledge about FAW in different areas of the Punjab (Pakistan). With the help of a pre-set questionnaire, data was collected by surveying farmers of different socio-economic conditions and from different locations. The socio-economic profile of maize growers might influence their perceptions regarding fall armyworm (FAW). The findings from this study will assist experts to develop a better pest management strategy and support other stakeholders to achieve sustainable control of FAW. Additionally, these results will provide guidance for future area-specific research on FAW management approaches.

## 2. Materials and Methods

### 2.1. Study Area

**Figure 1:**
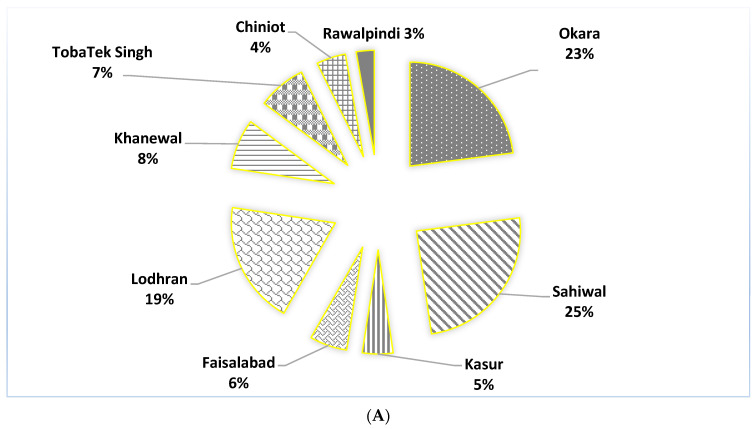

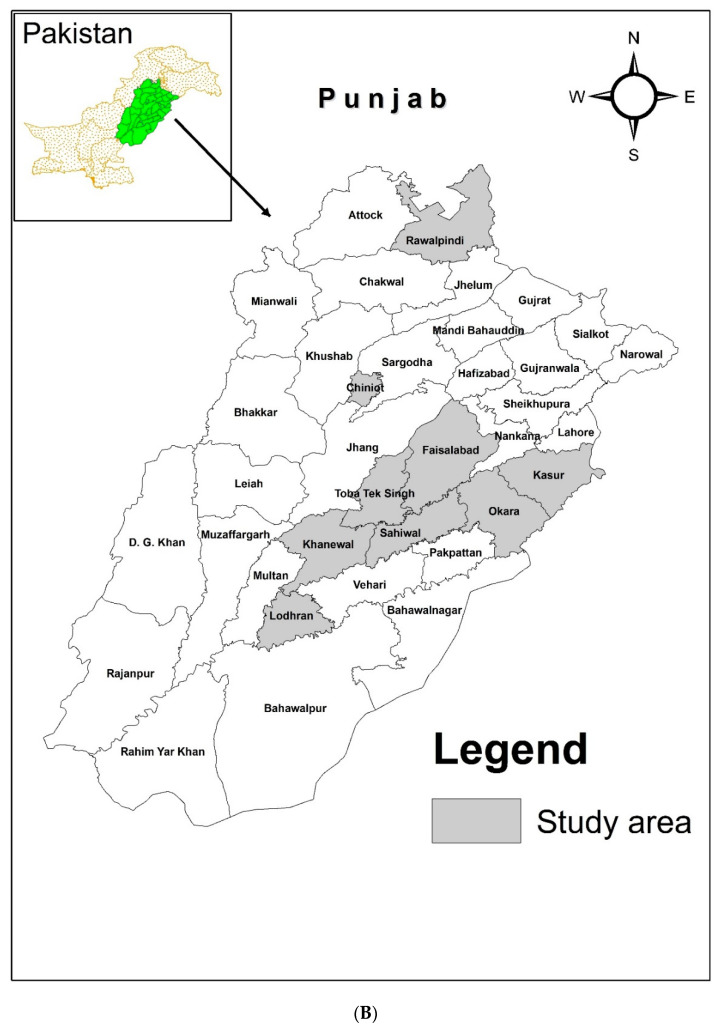
(**A**) Percentage shares of total surveyed area across nine districts of Punjab. (**B**) The surveyed districts of Punjab (Pakistan).

The study was conducted in the Punjab province of Pakistan due to its major share in maize crop production. About 80% of the hybrid maize produced in Pakistan is grown in Punjab. The selected nine districts were surveyed on the basis of maize crop density and their contribution in the national productivity of maize. Across these districts, the total maize surveyed area was 5767.5 acres. Moreover, district-wise percentage area is in Figure 1A.

The surveyed districts included Okara, Sahiwal, Kasur, Faisalabad, Lodhran, Khanewal, Toba Tek Singh, Chiniot and Rawalpindi (Figure 1B). The farmers were included in the survey regardless of their farm size and utilization of maize as fodder or a cash crop. The data regarding maize area and production from selected districts were collected through authentic sources [17,18], as shown in Table S1. The mean annual temperature of the surveyed districts ranged from 30.89 °C to 33.19 °C, with mean precipitation of 21.74 mm to 23.74 mm, as per Pakistan Meteorological Department. Details [25,26] are given in Table S1.

### 2.2. Survey Tools

A questionnaire was designed (in English) on the basis of some previous studies [22,23] and used as a survey tool to gather the required information. During the survey, the local language was adopted for the convenience of the respondents. Most of the questions had multiple choice options and a few were dichotomous (yes or no). The questionnaire was divided into four parts according to the information required, regarding socio-economic status, particulars about the crop, awareness about pests of maize and knowledge about FAW. Part-I includes the following: Socio-economic characteristics of farmers regarding age, education, type of farming profession, farming experience, total landholding and ownership details. Part-II: Information about maize crop cultivation, such as area, experience level, purpose of the crop, selection of growing seasons, seed type and its source. Part-III: Farmers’ awareness about insect pests of maize, pest management, information sources and direct or indirect contact for technical guidance. Part-IV: The recognition of fall armyworm, damage symptoms and levels, its spread, seasonal abundance and information about vulnerable parts, growth stages of maize, intensity of attack and any possible future threats.

### 2.3. Data Collection

The survey was carried out from February to October 2022. Before conducting a regular survey, the questionnaire had been pre-tested in one district. After some modifications in the questionnaire, 20–25 maize growers from each district were selected at random and interviewed for data collection. The participation of the respondents (208 farmers) was purely on a voluntary basis, but informed consent was sought before data collection by providing the objectives of our study. The survey was carried out using face to face interview in Urdu or the local language of the farmer, followed by the completion of the developed questionnaire. Each interview with a farmer was completed within one hour. Various visual aids, like picture or live specimens (or both), were also used to assist the respondent. Pictures or live specimen included different life stages of the insect and FAW damage symptoms on maize plant. These visual aids helped to determine the farmers’ understanding of FAW identification and damage [22,23,27].

### 2.4. Statistical Analysis

Statistical software of IBM SPSS Statistics 20 was used for descriptive and inferential analysis. Descriptive statistical analysis (frequencies and percentages) was carried out to summarize the socio-demographic data for understanding some specific attributes of the surveyed population. Likewise, inferential statistics and logistic regression were used to estimate the effect of different socio-economic factors on the overall understanding of farmers of fall armyworm. Multinomial logistic regression was performed to identify the factors that significantly influence farmers’ knowledge and perception of fall armyworm.

## 3. Results

### 3.1. Socio-Economic Characteristics of Farmers

The majority of the farmers surveyed (53%) belonged to middle-aged group (31–50 years). About 24% of the farmers fell under younger age group (20–30 years), and the remaining 23% of the respondents were older (≥51). Data analysis revealed that 16.8% of the farmers were illiterate, while 83.2% of the farmers were literate. Among the literate respondents (83.2%), the majority (54.3%) of respondents had completed their education above matriculation (>10 years of schooling), whereas the remaining 28.9% of the farmers had undergone primary to middle (5–8 years of schooling) school education (Table 1).

When asked whether they were farming as a part-time or full-time activity, 26% of the respondents reported that farming was not their primary income source, while 74% of the respondents were directly relying on farming for their livelihood. About 60.1% of the farmers have been practicing agriculture since birth (highly skilled) and 51.4% of the respondents were larger farmers with a landholding of more than 25 acres. Nearly 21.6% of the farmers owned their farm, whereas 21.2% of the respondents cultivated crops on rented land. Only 4.8% of the farmers cultivated land through mutual arrangements (personal and rental), while 52% of the respondents had mixed types of land ownership (Table 1).

### 3.2. Maize Crop Cultivation

The results further showed that most of the maize growers (37.5%) possessed medium landholdings (5–12.5 acres), while 26.9% of the maize farmers had very large farm area (≥25 acres). About 16.8% were recorded as large farmers (12.5–25 acres), whereas 18.8% farmers grew maize crop on an area of less than five acres (Table 2).

As far as experience of raising maize crop is concerned, 18.8% farmers were at a beginner level (1–10 years), and 16.8% of the farmers were at an intermediate level (21–30 years). Most (37.5%) respondents fell into the skilled category (11–20 years of maize cultivation), while 26.9% of the respondents were highly skilled in raising maize (Table 2).

Maize was planted as a multipurpose crop, mostly used as fodder, silage, for seed multiplication and as a cash crop. Approximately 31.3% of the farmers raised maize as a fodder crop for their cattle. As a cash crop, maize was grown by 30.3% of the maize growers and 26.4% of the respondents grew maize as a mixed-purpose crop, i.e., as fodder and a cash crop. The lowest number of farmers (12%) harvested maize crops for other purposes, which included seed production, silage, etc. (Table 2).

The majority of farmers (70.7%) sowed maize crops throughout the year by planting in both spring and autumn seasons. As a spring crop, maize was raised by 12% of the farmers only, whereas 17.3% of farmers raised a single crop during the autumn season (Table 2).

Only 5.8% of growers relied on seeds from open-pollinated varieties (OPV), while the majority of farmers (55.3%) used the latest hybrid seed. At the same time, 38.9% of the famers used both hybrid and OPV seeds (Table 2). Farmers obtained maize seed from different sources—about 83.2% of the farmers purchased seed from local dealers and traders. Approximately 3% of the farmers used their own seed (OPV), and 3% of the farmers obtained seed directly from seed companies. Some farmers (11.1%) availed of both sources of seed (produced at their farm or purchased from local dealers) (Table 2).

### 3.3. Awareness of Maize Insect Pests, Their Management Practices and Sources of Information

#### 3.3.1. Awareness of Maize Insect Pest and Their Management Practices

About 38.9% of the growers could identify FAW, while 31.7% could identify maize borers only. The results further indicated that 70.6% of the farmers were acquainted with Lepidopteran infestation on maize crops. In case of the shoot fly, only 14.4% of the respondents could recognize it. About 15% of the farmers reported that some other pests (termite/sucking) also damage maize crops (Table 3).

Pest management practices using chemicals were mainly adopted by 86% of the farmers, while the remaining 14% reported that they never used any chemicals. Among those who used chemicals, some farmers applied chemicals through foliar spraying (25%), while others (23.1%) used granular formulations to manage the insect pests on maize crop. However, 38% of the respondents opted for both techniques (spray and granular applications) in an alternate fashion to reduce the pest population (Table 3).

#### 3.3.2. Source of Agricultural Information

The majority of the farmers (56.7%) had direct contact with technical experts, like the Agriculture Department of Punjab (Govt. of Pakistan)/Pesticide Industry’s representatives, whereas 52.4% actively participated in farmer meetings to learn new techniques regarding crop cultivation and pest management (Table 3). For the acquaintance with FAW, the results indicated that most of the farmers (46%) observed the pest without any prior information from other stakeholders, whereas 16.3% heard about it for the first time from pesticide dealers or fellow farmers. In total, 13.5% of the respondents came to know about FAW by technical experts (Agriculture Department of Punjab/Pesticide Industry’s representatives). Lastly, around 23.5% of farmers had no information about it (Table 3).

### 3.4. Awareness of FAW Identification and Damage

About 60% of the farmers could not identify the larvae of fall armyworm, whereas only 39% were able to identify them. However, many (61%) farmers could identify the damage symptoms of FAW on maize plants. The majority of farmers (72.1%) were familiar with the fact that the larva is the most damaging stage of the fall armyworm, but 13.5% of the respondents had no idea about the most damaging stage of the insect. Only 14.4% of farmers believed that adults (moth) of FAW are responsible for direct damage to crops (Table 4).

### 3.5. Farmers’ Perception of FAW

Around 47.6% of the farmers did not know about the status of the fall armyworm—whether it was an established, sporadic or a minor pest—whereas 27.9% of the respondents tended to perceive FAW as an established pest. Almost 12% of the surveyed farmers considered FAW a minor pest, and 12.5% of the farmers ranked it as a sporadic pest (Table 4).

When farmers were asked about the spread of FAW in Pakistan, most (43.3%) believed that FAW was a natural phenomenon, some (15.4%) said that it spread from India, while others (15.4%) supposed that its source country was America. Approximately 26% of the respondents had no idea about its source of spread (Table 4).

Generally, farmers were not aware of the peak season for FAW incidence on maize crops. Some farmers (21.2%) reported FAW attacks on maize during both seasons (spring and autumn), whereas 40% of the respondents had no idea about its severity in relation to season. About 24% of the farmers assumed that FAW incidence was high during the spring season, while 33.7% of the farmers reported that incidence of fall armyworm was at its peak during the autumn season (Table 4).

### 3.6. Farmers’ Perception About Maize Crop Interaction with FAW

Approximately 15% of the farmers had no awareness of the most vulnerable phase of maize crops to FAW. About 35.6% of the farmers recorded vegetative stage, 21.2% farmers considered reproductive phase and 27.9% of the farmers reported both stages as vulnerable to FAW’s attack. Leaves were considered favorite part for larval feeding by 37% of the farmers. Around 32.3% of the farmers had no idea about the preferred part of maize for FAW. Similarly, 37.5% of the farmers did not know about the FAW larval feeding time, 26% of the respondents assumed that larva cause damage during daytime, whereas 37.5% of the farmers reported night as the feeding time of larvae (Table 4).

### 3.7. Previous Year Attack Intensity and Its Management

Nearly 50% of the surveyed farmers claimed that previous year’s attack of FAW was not as severe and that it was a low-intensity attack. But the previous year’s attack was managed well by 85.6% of the farmers. Only 14.4% of the respondents reported their crop was not managed properly. Interestingly, 11.1% of the maize growers did not consider FAW a future threat, 41.3% of the growers had no comments about future threat of FAW and 47.6% of the farmers considered FAW as a future threat. Among them, 35.1% of the respondents considered FAW a future threat to maize crops, whereas only 12.5% of the respondents considered it a future threat to other crops (Table 4).

### 3.8. Multinomial Logistic Regression

The effect of different predictor variables (age, education, type of farming and farming experience) on farmers’ awareness about FAW is given in Table 5. First of all, farmers were asked about the recognition of different pests (fall armyworm, shoot fly and other pests) considering maize borers as a reference category. Farmers across the different age groups could differentiate between FAW infestation and no attack of FAW on maize crops. Full-time farming showed a significant positive effect on farmers’ recognition of shoot fly (β = 1.538, SE = 0.669) and other pests (β = 1.71, SE = 0.656). The type of farming profession had an impact on farmers’ preferred source of information about FAW, which is statistically significant when compared to the reference category (self-observation). The respondents’ best choice was self-observation of FAW and they did not rely on technical experts, pesticide dealers or fellow farmers (β = −2.184, SE = 0.069). Different levels of farming experience also showed significant association with farmers’ source of information about FAW. Farmers were less likely to depend on technical experts (β = −0.523, SE = 0.204) compared to the self-observation (reference category). The negative sign showed that farmers prefer self-observation.

Statistical analysis showed that farmers with higher level of education (>10 years of education) tended to perceive FAW as minor pest (β = 0.808, SE = 0.383) compared to the do not know category (reference category). Farmers with full-time farming professions have a strong perception that FAW is a sporadic pest (β = −18.016, SE = 0.000), while part-time farmers tended to perceive it as a minor pest (β = −2.903, SE =1.116) compared to the reference category. The associations were significant, with negative signs indicating that their perception had a lower certainty level.

In case of farmers’ perception about the peak season of FAW, four options were given: spring, autumn, both seasons and do not know. Statistical analysis revealed that, overall, farmers had no exact idea of the seasonal peak traffic of FAW. But across all categories of farming profession, the majority of farmers perceived the highest peak of FAW activity to occur in the spring season (β = −1.224, SE = 0.615), compared to the do not know category (reference category). Farmers with a notion of the spring season as peak activity season of FAW with the negative sign in the coefficient indicated that their perception is significantly doubtful. Farmers’ perception about the most vulnerable crop phase to FAW larvae was categorized as vegetative, reproductive, both and do not know. The vegetative phase was used as a reference category and results showed that as education level of farmer increases (matriculation and above) the farmer believed that reproductive phase (β = 0.599, SE = 0.293) was more vulnerable to FAW. The do not know category (β = 0.526, SE = 0.249) indicated that farmers’ certainty levels were very poor regarding the vegetative and reproductive phases being the most susceptible stages of the maize plant.

As the education level of farmers increased, farmers had weaker perception about maize plant part preference by FAW larvae. Leaf preference (β = −0.562, SE = 1.038) and other parts of maize plant were (β = −0.889, SE = 0.288) compared with the do not know as reference category. Although this association was statistically significant, the negative sign indicated farmers’ uncertainty about maize plant part preference by FAW larvae.

Farmers with more farming experience tended to perceive FAW as a future threat to maize (β = −1.085, SE = 0.406) and threat to other crops (β = −1.281, SE = 0.437) as well, whereas the farmers with the least experience responded as Do not know (β = −0.803, SE = 0.402) when compared to reference category of no threat. However, significant results with a negative sign indicated that the majority of farmers did not perceive FAW as a future threat.

The results of multinomial logistic analysis about the effect of different predictor variables—size of landholding, nature of land possession, maize crop area and maize farming experience—on farmers’ perception about FAW are given in Table 6. The results indicated that there was significant association between farmers’ experience with maize crop and subsequent knowledge about pests of maize compared to (reference category) maize borers. As their experience increased, they had better knowledge of other pests (β = 0.665, SE = 0.331). The different nature of landholding (mutual, personal, leased/rented and mixed) did not have any effect on farmers’ perception about pest status of FAW (Table 6) when compared to the reference category (“do not know”). Only the established pest category showed statistically significant association (β = −0.412, SE = 0.211) for mutual landholding category, but the negative sign indicated farmers’ weaker perception.

In the case of maize crop area, the results showed that farmers with a small crop area generally considered FAW an established pest (β = 0.933, SE = 0.386), whereas farmers with medium crop area generally considered it a sporadic pest (β = 1.119, SE = 0.496) in comparison to the reference category (do not know). As the maize farm size increased, the farmers had a perception that the most preferred part for larva feeding was the leaf (β = −0.745, SE = 0.365) in comparison to the reference category. The negative sign indicated poor certainty levels of farmers about leaf as most preferred feeding part by FAW larvae.

Maize growers with large farm size had a perception that nighttime (β = 0.825, SE = 0.402) was the most preferred feeding time of FAW larvae, and the farmers with very large farm size responded as do not know (β = 1.161, SE = 0.440) in comparison to the reference category (feeding at daytime). The data analysis verified that experienced farmers had better perception about the feeding of FAW being nighttime (β = −0.765, SE = 0.320), whereas the most experienced farmers responded as do not know (β = −1.148, SE = 0.371) when compared to the ‘daytime’ as reference category. Interestingly, although maize farming experience and farmers’ perception about feeding time of FAW was significantly associated but negative signs showed that most of the farmers believed daytime as the preferred feeding time for FAW larvae.

Farmers with larger size of maize farms reported severe FAW attack (β = 1.167, SE = 0.457) during the previous year compared to the low-intensity attack category, and this association was statistically significant. Maize farmers with more experience tended to perceive FAW as a future threat to maize (β = −0.814, SE 0.418) in comparison to the reference category. Their perception had no certainty, as there is a negative sign with the value.

In Table 7, the effect of some predictor variables (purpose of maize crop, maize season availed, type of maize seed used and source of maize seed) on farmers’ perception about FAW is shown. The type of maize seed (hybrid or OPV) used by the farmers did not improve farmers’ knowledge about insect pests of maize, even the shoot fly (β = −0.926, SE = 0.422). Similarly, seed procurement sources had no impact on farmers’ awareness about FAW. They even responded as do not know (β = −0.725, SE = 0.309) about fall armyworm personally observed. Farmers growing maize for different purposes showed different opinions about the status of FAW as pests or established pests (β = −0.417, SE = 0.201). However, a statistically significant association was observed between different purposes of maize crop and farmers’ perception about FAW as pest. However, the coefficient values with negative sign showed an uncertainty about the pest status of FAW. The farmers who grew maize for both seasons assumed that FAW’s severity was observed in both seasons. (β = −1.002, SE = 0.355). The negative sign indicated the uncertainty in their knowledge.

In case of seed procurement methods, poor certainty was shown—some farmers responded about leaf (β = 0.855, SE = 0.435) and farmers who used mixed source of seed (own and dealers) assumed that FAW feeds on other parts (β = 1.056, SE= 0.443) rather than leaves compared to the reference category (do not know). Farmers’ knowledge about the most vulnerable phase of maize plant was not influenced by the farmers’ choice of maize season. They responded as do not know (β = −0.576, SE = 0.264) about the most vulnerable stage of the plant. Similarly, the source of the seed also influenced the farmers’ perception about the preferred feeding time of FAW larvae—they responded as nighttime (β = −1.109, SE = 0.356) and do not know (β = −0.900, SE = 0.332). The results indicated a statistically significant association, but the uncertainty, indicated by a negative sign, suggests that seed procurement from different sources may have influenced farmers’ perception about the feeding time of FAW. Non-technical personnel, such as pesticide dealers, may have shaped farmers’ perception that daytime was the preferred larval feeding time, which is actually the opposite. The farmers’ perception about the feeding preference of FAW larvae was affected by the purpose of the maize crop sown. They considered other plant parts (β = 0.624, SE = 0.223) as the most preferred feeding spots compared to leaves and the reference category (do not know).

Our results showed that farmers growing maize in both seasons were more likely to use the granular formulation of insecticides (β = −0.820, SE = 0.290) compared to the as reference category using no insecticides. The results were significant, but there is some uncertainty of the farmers indicated by the negative sign. When farmers were asked about the severity of FAW infestation during the previous year, those growing maize for different purposes—such as switching from fodder to mixed or other/seed purpose crop—believed that a severe attack (β = 0.512, SE = 0.240) of FAW had occurred in the previous year compared to the reference category of low-intensity attack.

Most of the farmers growing maize during one or both seasons reported that they do not know (β = −0.521, SE =0.273) about severity of FAW infestation in the previous year compared to the reference category (low-intensity attack). The negative sign showed uncertainty among farmers on this matter, and the association was almost significant. Across all sources of maize seeds, farmers significantly reported (β = 0.930, SE = 0.302) no FAW attack during the previous year. Different sources of maize seed used by the farmers had a significant effect on their prediction of the FAW threat to maize (β = −1.025, SE = 0.378) and do not know (β = −0.689 SE = 0.317) compared to the reference category (no threat).

## 4. Discussion

The current findings shed light on farmers’ perception and their understanding of different factors related to FAW affecting maize crop in the Punjab province of Pakistan. Demographic and socio-economic factors significantly impacted the working efficiency and learning ability of the individuals. Age is a crucial factor which governs the skills and work efficiency of farmers in dealing with this new maize pest. As the age of farmers increased, they had better knowledge about farming and FAW management practices [9]. The current study demonstrated that middle-aged farmers had better information about the severity of FAW in the previous cropping season. The dominance of the middle-aged group (31–50 years) indicated that the respondents were actively involved in maize farming. The same results regarding age group and FAW perception of farmers have been reported by many earlier studies [28,29]. Generally, farmers in this age group have been defined as the active farming class [30].

Our results did not establish any relationship between levels of education and the knowledge of farmers about the fall armyworm. Apparently, formal education had no impact on farmers’ perception about FAW’s status as a pest, the vulnerability of crop stages or the preferred plant parts attacked by FAW. In another study, Canico et al. [31] indicated that education did not improve farmers’ understanding of FAW. This situation seems to be a strong reason for the invasiveness of FAW, as farmers have no experience with this new pest. Generally, despite poor or no education, farmers had better ability and work efficiency with more farming experience [32]. Our study showed that more than 50% of the farmers had obtained high school level education or above. These findings are in line with the outcomes of the previous studies, indicating a widespread trend of acquiring formal education [22]. Moreover, they also associated farmers’ education with better understanding of FAW’s status and its management through pesticides. In the current study, we also observed that more than 70% of the respondents were directly relying on farming as a full-time profession [23]. Consequently, they had a better understanding of different maize pests. Farming experience usually enhances farmers’ ability and performance in the field, which is mostly associated with higher crop productivity [33]

Farmers generally seek information about farming practices and their management of FAW from various local sources, including fellow farmers [34]. However, these sources are not always authentic. In our study, the majority of farmers believed in self-observation regarding FAW, so their understanding of FAW was limited. Such behavior of farmers was also reported in previous studies, suggesting that famers learn through self-observation and intuitive knowledge [32,35]. The majority of farmers that opted for farming as a main source of income were always more vigilant in managing different pest problems in maize, including FAW, based on their knowledge and personal experience [36]. Other studies reported different sources of information, such as fellow farmers, media and extension workers, etc. [24], for technical guidance regarding farming practices.

The present studies showed that the majority of farmers had been practicing agriculture since birth (Highly skilled), had farming experience of more than 10 years and possessed more than 25 acres of land. We also noticed that more experience in maize farming improved farmers’ knowledge about different insect pests of maize and that they were able to differentiate among severely, less severely and not infested maize fields. But even some experienced farmers were unable to understand the behavior of this alien pest. If available, such information usually helps farmers in adopting timely pest management activities [31]. Large farm size, land ownership and farming experience may encourage farmers to invest more in different farm practices and the adoption of modern technologies to manage insect pests of maize including FAW [37].

Although some studies have reported that maize cultivation is usually practiced on a small scale, with landholdings of less than two hectares globally [38], most maize growers in our survey possessed medium landholdings for maize crops. Most farmers grow maize for personal consumption or business purposes [39]. Our study indicated that almost 60% of farmers had sown maize for domestic (fodder crops) and business uses (cash crops). Maize farm size also had an impact on farmers’ interest in learning more about FAW. Farmers who practiced maize farming on a smaller scale and for fodder purposes only had little knowledge about FAW. Therefore, they were not using any specific formulation of insecticide, granular or liquid, for its management, whereas farmers who raised maize as a cash crop and on a larger area generally managed FAW and other insects using insecticides. Principally, management of pests is directly related to knowledge about its damage potential and biology [23]. Furthermore, most farmers considered chemicals as an economic and effective choice for the rapid control of FAW [9]. We also concluded that respondents whose primary source of income was farming usually prefer chemicals to manage insect pests like FAW [40].

Our studies further revealed that farmers who grew maize on larger areas but were not directly involved in farming practices had poor knowledge about the status of FAW as pest. Hence, we can say that whether maize farming was on small or larger scale, for any purpose, had no impact on farmers’ knowledge about FAW, its status as pest, preferred feeding time or part of the maize plant it attacks. However, farmers were aware of the severe attack of FAW during the previous year, and this has also been reported in some other studies [6,24].

According to our study, the majority of farmers were raising maize crops twice a year (autumn and spring) by purchasing hybrid seeds from local markets and were in the habit of seeking agricultural advice from different sources, such as pesticide dealers, technical experts and farmer meetings. This is a common practice among farmers and has been reported in many other studies [24,41]. We also observed that farmers had better information about different insect pests of maize, except for FAW. While farmers were able to identify the damage symptoms of FAW on the foliage and whorls of maize, their ability to identify the larvae was poor. Farmers’ perception about FAW may be influenced by the non-technical dealers. They perceived that daytime was the preferred larval feeding time, whereas studies have reported that noctuid larvae prefer to feed at night [42].

Host plant availability and optimal ecological conditions support the reproduction and survival of FAW [43], enabling it to damage maize crops throughout the year. Some previous studies have shown that cropping patterns and varietal differences also play a vital role in the severity of FAW [44]. As there is no diapause in FAW [45], its presence on maize in both seasons has led farmers to perceive FAW as an established pest. However, they were not sure about its severity in the previous year. Moreover, their knowledge of its peak infestation season, proper feeding time for larvae, and the most vulnerable phase of the maize crop was also poor. Furthermore, they had no concern about its origin or spread in Pakistan. Such limited knowledge made them less competent to decide about the status of FAW, its proper control as a pest and as a future threat to maize crops. The invasiveness of FAW, its ability to survive across continents, its adaptation to diverse climatic conditions and the history of substantial maize yield losses calls for farmers and policymakers to devise effective management strategies in Pakistan to prevent future invasions [31].

The fall armyworm’s dramatic spread across continents, along with its voracious feeding habits, have made it a global threat to maize growers. The severe and rapid losses to maize crops amounting to millions of dollars across the world demand urgent action to manage this pest. Regional joint ventures are required to combat trans-boundary pests like FAW for sustainable management. There are two important pillars of successful pest management: control strategies (plaining and policies) and human activities (execution). Generally, pest control strategies alone are focused on and developed by researchers, while the attitude and execution knowledge of farmers are often ignored. Such negligence seems to be the main hurdle in the acceptance of new technologies and the consequent failure of pest control activities. As there are no previous data available in Pakistan regarding farmers’ perception and knowledge about fall armyworm, there is an urgent need to fill this gap. Such empirical studies will pave the way for managing any invasive pest across the country. These findings will further help policymakers to revise extension approaches, pest management protocols, and research programs to address farmers’ needs. To achieve this, local stakeholders, including the farming community and policymakers, must agree on any initiatives related to pest problems. Hence, it can be concluded that updating farmers’ knowledge about FAW will enable farmers to take time to make appropriate decisions regarding its effective management.

## 5. Conclusions

Our study revealed that farming is the primary profession of the respondents of our study in the Punjab province of Pakistan, particularly the middle-aged group that is actively involved in maize farming. Farmers with better experience grow maize for domestic and commercial purposes in both seasons, but they have poor knowledge about fall armyworm, its lifecycle, peak activity periods and control measures. Generally, education levels do not improve their knowledge about FAW, as most of the interviewed farmers believe and learn through self-observation and experience. Farmers with larger landholdings and more experience were more likely to invest in pest management practices through chemical control, yet comprehensive knowledge about FAW remained inadequate. Successful pest management of FAW requires eco-friendly protocols and improved farmers’ knowledge. These goals are achievable through interactive research and collaborative work of different stakeholders, with the involvement of policymakers, farming community and the agricultural extension department.

## Figures and Tables

**Table 1:** Scio-economic characteristics of maize farmers of Punjab (Pakistan).

Particulars	Categories	Frequency	%
Age (yrs.)	(Young) 20–30	50	24.0
	(Middle age) 31–50	110	52.9
	(Old) 51 and above	48	23.1
Qualification	(Illiterate) None	35	16.8
	Primary to middle	60	28.9
	Matric and above	113	54.3
Type of farming profession	Full time	154	74.0
	Part Time	54	26.0
Farming experience (yrs.)	(Beginners) 1–10	28	13.5
	(Skilled) 11–20	24	11.5
	(Intermediate) 21–30	31	14.9
	(Highly skilled) Since birth	125	60.1
Landholdings (acres)	(Small) Less than 5	10	4.8
	(Medium) 5–12.5 acres	49	23.6
	(Large) 12.5–25 acres	42	20.2
	(Very Large) 25 and above	107	51.4
Nature of holding/farm ownership	Mutual	10	4.8
	Personal	45	21.6
	Lease/Rented	44	21.2
	Mixed	109	52.4

**Table 2:** Particulars about maize crop area and cultivation in Punjab (Pakistan).

Particulars	Categories	Frequency	%
Maize farm area (acres)	(Small) Less than 5	39	18.8
	(Medium) 5–12.5	78	37.5
	(Large) 12.5–25	35	16.8
	(Very Large) ≥ 25	56	26.9
Maize cultivation experience (yrs.)	(beginners) 1–10	39	18.8
	(Skilled) 11–20	78	37.5
	(Intermediate) 21–30	35	16.8
	(Highly skilled) Since Birth	56	26.9
Purpose of maize crop	Fodder	65	31.3
	Cash crop	63	30.3
	Fodder + cash crop (both)	55	26.4
	Silage + seed	25	12.0
Selection of maize season	Spring	25	12.0
	Autumn	36	17.3
	Both	147	70.7
Type of maize seed	OPV	12	5.8
	Hybrids	115	55.3
	Mix	81	38.9
Source/procurement of maize Seed	Own seed	6	2.9
	Trader/dealer	173	83.2
	Company	6	2.9
	Mix	23	11.1

**Table 3:** Farmers’ awareness of insect pests of maize, their management and sources of information in Punjab (Pakistan).

Particulars	Categories	N	%	Particulars	Categories	N	%
Insect pests of maize and their identification	Maize borers	66	31.7	Contact with experts (research or extension services)	Yes	118	56.7
	Fall armyworm	81	38.9		No	90	43.3
	Shoot fly	30	14.4	Participation in farmer meetings/trainings	Yes	109	52.4
	Others (termites/sucking)	31	14.9		No	99	47.6
Pest management choices	Foliar spray	52	25.0	How did you hear about FAW	Yourself	97	46.6
	Granular	48	23.1		Technical experts (extension Dep./pesticide industry rep.)	28	13.5
	Both	79	38.0		Dealer/fellow farmers	34	16.3
	Nothing	29	13.9		Do not know	49	23.6

**Table 4:** Awareness about identification of FAW, its damage and interference with maize crop in Punjab (Pakistan).

Particulars	Categories	N	%	Particulars	Categories	N	%
FAW identification	Yes	83	39.9	Most vulnerable phase of maize plant to FAW	Vegetative	74	35.6
	No	125	60.1		Reproductive	44	21.2
FAW damage identification	Yes	127	61.1		Both	58	27.9
	No	81	38.9		Do not know	32	15.4
Most damaging stage of Insect	Larvae	150	72.1	Favorite part of maize plant for FAW	Leaf	77	37.0
	Adult	30	14.4		Others	64	30.8
	Do not know	28	13.5		Do not know	67	32.2
Status of FAW	Established Pest	58	27.9	FAW larval feeding time	Day	54	26
	Sporadic pest	26	12.5		Night	76	35.6
	Minor pest	25	12.0		Do not know	78	37.5
	Do not know	99	47.6	Previous year attack intensity	Do not know	34	16.3
From where did it spread	India	32	15.4		No attack	39	18.8
	America	32	15.4		Low-intensity attack	104	50.0
	Natural	90	43.3		Severe attack	31	14.9
	Do not know	54	26.0	Previous year control of FAW	Controlled	178	85.6
Season of FAW	Spring	50	24.0		Not controlled	30	14.4
	Autumn	70	33.7	Future threat/fate of FAW	Do not know	86	41.3
	Both	44	21.2		Threat to maize crops	73	35.1
	Do not know	44	21.2		Threat to other crops	26	12.5
					No threat	23	11.1

**Table 5:** Impact of socio-economic factors of maize farmers on perception of FAW in Punjab.

		Predictors			
Variables		Age	Education	Type of Farming	Farming Experience
Can you recognize different pests of maize?					
	Fall armyworm	−0.042 (0.269)	0.105 (0.243)	1.08 (0.567)	0.059 (0.16)
	Shoot fly	0.319 (0.374)	−0.059 (0.330)	1.538 (0.699) *	−0.058 (0.788)
	Others (termites/sucking)	0.100 (0.369)	−0.121 (0.328)	1.71 (0.656) **	0.094 (0.219)
	Maize borers	Reference category			
From where did you first hear about FAW?					
	Technical experts	−0.188 (0.357)	0.031 (0.32)	−1.512 (0.837)	−0.523 (0.204) **
	Dealer/fellow farmers	−0.038 (0.319)	−0.162 (0.284)	−2.184 (1.069) *	−0.109 (0.204)
	Do not know	−0.105 (0.321)	0.063 (0.287)	0.777 (0.475)	−0.188 (0.191)
	Self-observation	Reference category			
FAW’s status as pest perceived by the farmer?					
	Established pest	−0.042 (0.289)	0.001 (0.263)	−0.833 (0.484)	−0.191 (0.171)
	Sporadic pest	−0.294 (0.376)	0.174 (0.339)	−18.016 (0.000) ***	−0.100 (0.223)
	Minor pest	0.409 (0.392)	0.808 (0.383) *	−2.903 (1.116) **	−0.086 (0.237)
	Do not know	Reference category			
From where did FAW spread to Pakistan?					
	India	0.048 (0.361)	−0.129 (0.313)	−0.507 (0.659)	−0.062 (0.221)
	America	0.356 (0.367)	0.273 (0.324)	−0.613 (0.606)	−0.297 (0.209)
	Natural	0.071 (0.278)	0.383 (0.251)	−0.573 (0.206)	0.035 (0.166)
	Do not know	Reference category			
In which season has the severity of FAW been observed?					
	Spring	0.204 (0.335)	−0.041 (0.310)	−1.224 (0.615) *	0.188 (0.205)
	Autumn	−0.151 (0.328)	0.299 (0.294)	−0.464 (0.497)	0.292 (0.186)
	Both	−0.222 (0.370)	0.047 (0.326)	−0.962 (0.626)	0.160 (0.212)
	Do not Know	Reference category			
Do you know about the most damaging stage of FAW?					
	Larvae	0.420 (0.312)	0.042 (0.268)	−0.596 (0.487)	−0.110 (0.180)
	Adult	0.177 (0.349)	0.536 (0.315)	−0.892 (0.566)	−0.067 (0.202)
	Do not Know	Reference category			
Farmers’ perception about the most vulnerable crop phase to larvae of FAW?					
	Reproductive	0.422 (0.321)	0.599 (0.293) *	−0.490 (0.582)	−0.189 (0.191)
	Both	−0.011 (0.458)	−0.085 (0.375)	−0.500 (0.877)	−0.476 (0.255)
	Do not Know	0.451 (0.276)	0.526 (0.249) *	0.131 (0.454)	−0.111 (0.163)
	Vegetative	Reference category			
Can you talk about the most preferred part of the maize plant for FAW larvae ?					
	Leaf	0.037 (0.277)	−0.562 (1.038) *	−0.169 (0.500)	0.149 (0.162)
	Other parts	0.322 (0.310)	−0.889 (0.288) **	0.691 (0.521)	0.105 (0.191)
	Do not know	Reference category			
Do you have any idea about the preferred feeding time of FAW larvae?					
	Nighttime	−0.141 (0.318)	0.351 (0.869)	0.056 (0.526)	−0.228 (0.188)
	Do not know	−0.178 (0.317)	0.113 (0.284)	−0.446 (0.568)	−0.031 (0.196)
	Daytime	Reference category			
What type of chemical formulation do you prefer to manage FAW?					
	Spray	0.378 (0.337)	0.257 (1.460)	−0.110 (0.656)	−0.342 (0.194)
	Granular	−0.166 (0.321)	−0.119 (0.286)	1.067 (0.545) *	0.169 (0.186)
	Both	0.555 (0.359)	0.068 (0.326)	1.153 (0.550) *	−0.370 (0.207)
	Nothing	Reference category			
Do you have any information about the intensity of FAW in maize crop last year?					
	Do not Know	−0.010 (0.900)	−0.077 (0.990)	−0.178 (0.566)	−0.224 (0.186)
	No attack	0.959 (0.366) **	−0.010 (0.317)	−0.660 (0.615)	0.089 (0.233)
	Severe attack	0.662 (0.345) *	−0.168 (0.639)	−0.590 (0.639)	−0.385 (0.205)
	Low-intensity attack	Reference category			
What was the perception of farmers about FAW as a future threat?					
	Do not know	−0.267 (0.437)	−0.467 (0.437)	−0.582 (0.661)	−0.803 (0.402) *
	Threat to maize crops	−0.245 (0.452)	−0.043 (0.454)	−0.667 (0.689)	−1.085 (0.406) **
	Threat to other crops	0.061 (0.533)	−0.109 (0.516)	−2.051 (1.001) *	−1.281 (0.003) **
	No threat at all	Reference category			

* Significant at *p* < 0.05, ** significant at *p* < 0.01, *** significant at *p* < 0.001; values in parenthesis show standard errors.

**Table 6:** Impact of land and farm characteristics on maize farmers’ perception of FAW in Punjab.

Variables		Predictors			
		Size of Landholding	Nature of Land Possession	Maize Crop Area	Maize Farming Experience
Can you recognize different pests of maize?					
	Fall armyworm	0.280 (0.294)	0.124 (0.188)	−0.135 (0.335)	0.103 (0.285)
	Shoot fly	−0.264 (0.419)	0.154 (0.275)	−0.299 (0.453)	0.597 (0.347)
	Others (termites/sucking)	−0.277 (0.417)	0.169 (0.294)	−0.466 (0.433)	0.665 (0.331) *
	Maize borers	Reference category			
From where did you first hear about FAW?					
	Technical experts	−0.565 (0.393)	0.359 (0.272)	0.148 (0.446)	0.035 (0.396)
	Dealer/fellow farmers	−0.486 (0.369)	0.428 (0.251)	−0.067 (0.396)	0.143 (0.335)
	Do not know	−1.187 (0.393) **	0.099 (0.238)	0.190 (0.421)	0.440 (0.306)
	Self-observation	Reference category			
FAW’s status as pest perceived by the farmer?					
	Established pest	0.197 (0.328)	−0.412 (0.211) *	0.933 (0.386) *	−0.410 (0.312)
	Sporadic pest	0.087 (0.417)	−0.390 (0.274)	1.119 (0.496) *	−0.483 (0.425)
	Minor pest	−0.322 (0.406)	−0.119 (0.288)	0.653 (0.505)	−0.359 (0.427)
	Do not know	Reference category			
From where did FAW spread to Pakistan?					
	India	0.343 (0.406)	−0.257 (0.258)	0.024 (0.435)	0.092 (0.348)
	America	0.015 (0.415)	0.095 (0.268)	0.201 (0.444)	0.129 (0.355)
	Natural	0.277 (0.310)	−0.094 (0.215)	−0.153 (0.335)	0.232 (0.257)
	Do not know	Reference category			
In which season has the severity of FAW been observed?					
	Spring	0.447 (0.376)	−0.037 (0.256)	0.340 (0.412)	−0.084 (0.323)
	Autumn	−0.140 (0.354)	0.363 (0.261)	0.371 (0.383)	−0.220 (0.280)
	Both	0.251 (0.401)	0.040 (0.269)	0.777 (0.454)	−0.502 (0.346)
	Do not know	Reference category			
Do you know about the most damaging stage of FAW?					
	Larvae	−0.219 (0.314)	0.318 (0.224)	0.349 (0.367)	−0.239 (0.275)
	Adult	0.160 (0.373)	−0.009 (0.241)	0.068 (0.411)	−0.042 (0.318)
	Do not know	Reference category			
Farmers’ perception about the most vulnerable crop phase to larvae of FAW?					
	Reproductive	−0.468 (0.360)	−0.013 (0.277)	0.471 (0.401)	−0.233 (0.330)
	Both	0.007 (0.466)	−0.151 (0.316)	−0.074 (0.543)	−0.054 (0.461)
	Do not know	−0.215 (0.303)	0.011 (0.199)	−0.237 (0.332)	0.383 (0.268)
	Vegetative	Reference category			
Can you talk about the most preferred part of the maize plant for FAW larvae ?					
	Leaf	0.146 (0.306)	−0.103 (0.193)	−0.745 (0.365) *	0.221 (0.316)
	Other parts	−0.390 (0.357)	0.110 (0.223)	−0.676 (0.408)	0.462 (0.337)
	Do not know	Reference category			
Do you have any idea about the preferred feeding time of FAW larvae?					
	Nighttime	0.243 (0.350)	−0.152 (0.252)	0.825 (0.402) *	−0.765 (0.320) *
	Do not know	0.563 (0.357)	−0.273 (0.250)	1.61 (0.440) **	−1.148 (0.371) **
	Daytime	Reference category			
What type of chemical formulation did you prefer to manage FAW?					
	Spray	−0.009 (0.306)	−0.284 (0.235)	0.364 (0.412)	−0.157 (0.335)
	Granular	0.454 (0.338)	0.105 (0.248)	−0.288 (0.397)	−0.463 (0.334)
	Both	0.658 (0.409)	0.188 (0.242)	0.003 (0.433)	−0.320 (0.374)
	Nothing	Reference category			
Did you have any information about intensity of FAW in maize crop last year?					
	Do not know	−0.367 (0.343)	0.098 (0.251)	0.333 (0.428)	−0.417 (0.361)
	No attack	−0.123 (0.398)	0.070 (0.250)	−0.338 (0.427)	0.552 (0.326)
	Severe attack	−0.446 (0.426)	0.086 (0.226)	1.167 (0.457) **	−0.466 (0.377)
	Low intensity	Reference category			
What was the perception of farmers about FAW as a future threat?					
	Do not know	0.297 (0.545)	0.106 (0.362)	0.143 (0.567)	−0.711 (0.61)
	Threat to maize crop	0.504 (0.562)	−0.090 (0.359)	0.585 (0.595)	−0.814 (0.418) *
	Threat to other crops	1.036 (0.653)	−0.007 (0.400)	0.471 (0.675)	−0.768 (0.517)
	No threat at all	Reference category			

* Significant at *p* < 0.05, ** significant at *p* < 0.01; values in parenthesis show standard errors.

**Table 7:** Impact of maize farming practices on farmers’ perception of FAW in Punjab.

Variables		Predictors			
		Purpose of Maize Crop	Selection of Maize Season	Type of Maize Seed	Source of Maize Seed
Can you recognize different pests of maize?					
	Fall armyworm	0.329 (0.191)	0.001 (0.270)	0.242 (0.321)	-0.321 (0.326)
	Shoot fly	0.347 (0.256)	−0.721 (0.334) *	0.926 (0.422) ***	0.302 (0.374)
	Others (termites/sucking)	0.050 (0.255)	−0.686 (0.333) *	0.205 (0.480)	0.401 (0.340)
	Maize borers	Reference category			
From where did you first hear about FAW?					
	Technical experts	−0.397 (0.276)	−0.278 (0.348)	-0.050 (0.433)	−0.068 (0.547)
	Dealer/fellow farmers	−0.204 (0.230)	0.556 (0.366)	-0.029 (0.374)	0.238 (0.381)
	Do not know	0.107 (0.217)	0.028 (0.303)	-0,091 (0.359)	0.725 (0.309) **
	Self-observation	Reference category			
FAW’s status as pest perceived by the farmer?					
	Established pest	−0.417 (0.201) *	0.641 (0.305) *	0.433 (0.335)	−0.555 (0.320)
	Sporadic pest	−0.026 (0.276)	0.059 (0.321)	0.637 (0.466)	−0.981 (0.727)
	Minor pest	−0.538 (0.273)	0.039 (0.343)	0.827 (0.450)	−1.251 (0.616) *
	Do not know	Reference category			
From where did FAW spread to Pakistan?					
	India	0.209 (0.247)	−0.105 (0.342)	0.607 (0.435)	−1.486 (0.705) *
	America	0.011 (0.254)	0.153 (0.366)	0.348 (0.417)	−0.771 (0.467)
	Natural	−0.074 (0.191)	−0.187 (0.266)	0.106 (0.308)	−0.025 (0.257)
	Do not know	Reference category			
In which season has the severity of FAW severity been observed?					
	Spring	0.047 (0.239)	0.550 (0.308)	0.035 (0.395)	−0.710 (0.377)
	Autumn	0.016 (0.221)	0.597 (0.290) *	0.138 (0.348)	−0.262 (0.284)
	Both	0.016 (0.254)	1.002 (0.355) **	−0.160 (0.423)	−1.006 (0.446) *
	Do not know	Reference category			
Do you know about the most damaging stage of FAW?					
	Larvae	−0.092 (0.206)	0.206 (0.306)	−0.177 (0.342)	0.192 (0.305)
	Adult	−0.028 (0.234)	−0.233 (0.334)	−0.473 (0.388)	−0125 (0.358)
	Do not know	Reference category			
Farmers’ perception about the most vulnerable crop phase to larvae of FAW?					
	Reproductive	−0.248 (0.221)	−0.139 (0.314)	−0.477 (0.382)	−0.544 (0.419)
	Both	−0.027 (0.327)	−0.072 (0.421)	−0.278 (0.544)	−0.010 (0.560)
	Do not know	0.248 (0.188)	−0.576 (0.264) *	0.037 (0.314)	0.403 (0.284)
	Vegetative	Reference category			
Do you have any idea about the preferred part of maize plant by larvae of FAW?					
	Leaf	−0.056 (0.204)	0.383 (0.259)	0.502 (0.339)	0.855 (0.435) *
	Other parts	0.624 (0.223) **	0.086 (0.287)	0.383 (0.373)	1.056 (0.434) **
	Do not know	Reference category			
Do you have an idea about the preferred feeding time of FAW larvae?					
	Nighttime	−0.066 (0.217)	−0.121 (0.296)	−0.199 (0.361)	−1.109 (0.356) **
	Do not know	−0.075 (0.218)	0.223 (0.312)	−0.558 (0.371)	−0.900 (0.332) **
	Daytime	Reference category			
What type of chemical formulation do you prefer to manage FAW?					
	Spray	−0.186 (0.233)	−0.575 (0.304)	−0.351 (0.399)	−0.325 (0.383)
	Granular	−0.350 (0.226)	−0.820 (0.290) **	−0.063 (0.380)	0.020 (0.349)
	Both	−0.272 (0.242)	−0.551 (0.337)	−0.325 (0.418)	−0.687 (0.517)
	Nothing	Reference category			
Do you have any information about the intensity of FAW in maize crop last year?					
	Do not know	0.129 (0.233)	−0.521 (0.273) *	−0.722 (0.403)	−0.225 (0.432)
	No attack	0.439 (0.235)	0.344 (0.435)	−0.342 (0.384)	0.930 (0.302) **
	Severe attack	0.512 (0.240) *	−0.295 (0.314)	−0.245 (0.429)	−0.243 (0.525)
	Low-intensity attack	Reference category			
What was the perception of farmers about FAW as a future threat?					
	Do not know	0.113 (0.290)	0.069 (0.443)	0.254 (0.428)	−0.689 (0.317) *
	Threat to maize crop	0.295 (0.301)	0.283 (0.456)	0.142 (0.457)	−1.025 (0.378) **
	Threat to other crops	−0.139 (0.368)	0.397 (0.535)	0.548 (0.580)	−1.005 (0.656)
	No threat at all	Reference category			

* Significant at *p* < 0.05, ** significant at *p* < 0.01, *** significant at *p* < 0.001; values in parenthesis show standard errors.

## Data Availability

All data generated or analyzed during this study are included in the article/Supplementary Material. Further inquiries can be directed to the corresponding authors.

## Supplementary Materials

The following supporting information can be downloaded at https://www.mdpi.com/article/10.3390/insects16040427/s1. Table S1: Area and production of maize in surveyed districts of Punjab.
