# Supplementary material for: Farmers’ Perception of Fall Armyworm (Spodoptera frugiperda) as an Invasive Pest and Its Management
Source: Insects. 2025 Apr 18;16(4):427. doi: 10.3390/insects16040427 (PMC12027892; doi:10.3390/insects16040427)
Supplement: Supplementary file 1 [file insects-16-00427-s001.zip › insects-3550632-supplementary.pdf]

Supplementary Data

Table S1: Area and production of maize in surveyed districts of Punjab

Districts	Maize Area(000) ha	Production (000)tons	Geo-position		Altitude(m)	Mean Temp (°C)	Annual Rainfall (mm)
**Okara**	372.8	1294.8	30.67772	73.10681	172	30.4	21.74
**Sahiwal**	221.0	702.9	30.8085	73.4594	179	31.25	22.35
**Kasur**	147.4	537.4	31.02021	73.85333	191	30.89	22.10
**Faisalabad**	121.1	315.1	31.41872	73.07911	175	31.87	22.80
**Lodhran**	117.4	319.0	31.72091	72.97836	179	30.98	22.16
**Khanewal**	95.9	300.5	31.14926	72.68323	167	31.79	22.74
**TobaTek Singh**	104.0	282.5	29.5339	71.63244	170.5	33.04	23.63
**Chiniot**	59.9	190.5	30.28642	71.93203	136	31.79	22.74
**Rawalpindi**	112.7	58.8	33.62606	73.07144	488	33.19	23.74
